# MiR-30b Attenuates Neuropathic Pain by Regulating Voltage-Gated Sodium Channel Nav1.3 in Rats

**DOI:** 10.3389/fnmol.2017.00126

**Published:** 2017-05-05

**Authors:** Songxue Su, Jinping Shao, Qingzan Zhao, Xiuhua Ren, Weihua Cai, Lei Li, Qian Bai, Xuemei Chen, Bo Xu, Jian Wang, Jing Cao, Weidong Zang

**Affiliations:** ^1^Department of Anatomy, Basic Medical Sciences College, Zhengzhou UniversityZhengzhou, China; ^2^Department of Anesthesiology, The Second Affiliated Hospital of Zhengzhou UniversityZhengzhou, China; ^3^Department of Anesthesiology, General Hospital of Guangzhou Military Command of People’s Liberation ArmyGuangzhou, China; ^4^Department of Anesthesiology and Critical Care Medicine, Johns Hopkins University School of Medicine, BaltimoreMD, USA

**Keywords:** Nav1.3, miR-30b, neuropathy pain, dorsal root ganglion, spinal cord

## Abstract

Nav1.3 is a tetrodotoxin-sensitive isoform among voltage-gated sodium channels that are closely associated with neuropathic pain. It can be up-regulated following nerve injury, but its biological function remains uncertain. MicroRNAs (miRNAs) are endogenous non-coding RNAs that can regulate post-transcriptional gene expression by binding with their target mRNAs. Using Target Scan software, we discovered that SCN3A is the major target of miR-30b, and we then determined whether miR-30b regulated the expression of Nav1.3 by transfecting miR-30b agomir through the stimulation of TNF-α or by transfecting miR-30b antagomir in primary dorsal root ganglion (DRG) neurons. The spinal nerve ligation (SNL) model was used to determine the contribution of miR-30b to neuropathic pain, to evaluate changes in Nav1.3 mRNA and protein expression, and to understand the sensitivity of rats to mechanical and thermal stimuli. Our results showed that miR-30b agomir transfection down-regulated Nav1.3 mRNA stimulated with TNF-α in primary DRG neurons. Moreover, miR-30b overexpression significantly attenuated neuropathic pain induced by SNL, with decreases in the expression of Nav1.3 mRNA and protein both in DRG neurons and spinal cord. Activation of Nav1.3 caused by miR-30b antagomir was identified. These data suggest that miR-30b is involved in the development of neuropathic pain, probably by regulating the expression of Nav1.3, and might be a novel therapeutic target for neuropathic pain.

**Perspective:** This study is the first to explore the important role of miR-30b and Nav1.3 in spinal nerve ligation-induced neuropathic pain, and our evidence may provide new insight for improving therapeutic approaches to pain.

## Introduction

The IASP (International Association for the Study of Pain) defines pain as an unpleasant sensory and emotional experience associated with actual or potential tissue damage, or described in terms of such damage (Merskey, 1979). The approximate prevalence of neuropathic pain in the gross population is 7–10% (Bouhassira et al., 2008; de Moraes Vieira et al., 2012) and remains extremely difficult to cure, mainly due to barely understood pathogenesis and a lack of well-defined molecular targets.

The voltage-gated sodium channels (VGSCs, Nav1.1–Nav1.9 and Nax) containing tetrodotoxin-sensitive (TTXS) channels and tetrodotoxin-resistant channels (TTX-R) are involved in the generation and propagation of action-potential (Casals-Díaz et al., 2015). Moreover, TTX-S Nav1.3 and Nav1.7, as well as the TTX-R Nav1.8 and Nav1.9 have been shown to implicate chronic neuropathic pain (Dib-Hajj et al., 2009). Nav1.3 is a subunit among the VGSCs, encoded by the SCN3A gene, and located on chromosome 2 (Estacion et al., 2010). SCN3A has a high expression in the central nervous system of embryos and newborns but is poorly expressed in adult rats (Estacion et al., 2010). Epilepsy (Guo et al., 2008; Vanoye et al., 2014), mental retardation (Bartnik et al., 2011), autism (Celle et al., 2013), and neuropathic pain (Chen et al., 2014) are perhaps caused by the aberrant expression of SCN3A. Nav1.3 is re-expressed in DRG neurons after peripheral nerve injury (Kim et al., 2001; Huang et al., 2014). Similarly, the level of Nav1.3 increases in lumbar dorsal horn neurons following SCI surgery (Hains et al., 2003; Lindia et al., 2005). In a previous study, the repression of Nav1.3 using Nav1.3-specific antisense (AS) oligodeoxynucleotide (ODN) blocked mechanical and thermal allodynia (Hains et al., 2005). However, the mechanism of altered Nav1.3 expression continues to perplex. It was reported that inhibition of the expression of NF-K B could prevent neuropathic pain by suppressing Nav1.3 re-expression in an L5-VRT model (Hains et al., 2004). Several studies focused on intrathecal lidocaine delivery to attenuate neuropathic pain through modulating Nav1.3 expression and reducing the activation of the spinal microglial (Zang et al., 2010).

Although studies have partly elucidated the mechanism of altered Nav1.3 expression, the entire, specific mechanism has not yet been made explicit. Non-coding RNA (ncRNA) regulating the expression of proteins has emerged as a target. In our study, we concerned on the relevant ncRNA that regulated the pain-related proteins. MicroRNAs (miRNAs) are endogenous ncRNAs from a single RNA precursor of 70–90 bases processed by a dicer enzyme to produce 19–25 mature nucleotides. They are responsible for the regulation of gene expression through the targeting of the gene 3′UTR (Monroig Pdel et al., 2014; Khan et al., 2015). MiRNAs are highly deregulated in diseases and might be a critical molecule for treatment, as they can be directly transfected into cells both *in vitro* and *in vivo* (Rajendiran et al., 2014; Yang et al., 2016), owing to the short nucleotide sequence (Griffiths-Jones et al., 2008).

Using Target Scan software, miR-30b, miR-96, mir-183, and miR-132 were predicted to highly relate to SCN3A. Furthermore, bioinformatics software showed that there are eight nucleotides that match miR-30b and SCN3A 3′UTR. In the present study, we focused on miR-30b, and we intended to verify whether miR-30b could regulate the expression of Nav1.3, as well as to explore the possibility that miR-30b could potentially alleviate neuropathic pain by changing the expression of Nav1.3 in DRG and the spinal cord

## Materials and Methods

### Animals

Male Sprague–Dawley rats (200–250 g), with food and water ad libitum, were housed in separate cages in a clean and open room with a stable and controlled temperature and a 12 h light–dark cycle. The rats were kept for at least 7 days before the operation. The procedures for the care and use of animals followed the recommendations and guidelines of the National Institutes of Health and were approved by Zhengzhou University Animal Care and Use Committee.

### Surgical Procedures and Drug Infusion

The rats underwent unilateral L5 SNL modification, as previously described (Fan et al., 2014). After the animals were anesthetized, the transverse process of the left L6 was removed to expose the L4 and L5 spinal nerves. After isolating the left L5 spinal nerve, a tight ligature was made with 3–0 silk, and the nerve was transected distal to this ligature. In the sham-operated group, the left L5 spinal nerve was separated but remained complete and unscathed with no ligature or transection.

Rats underwent intrathecal catheter implantation for drug delivery in the same manner as previously described (Liang et al., 2013). Briefly, under 2% isoflurane-induced anesthesia, a lumber laminectomy of the L5 vertebra was carried out and the dura was cut. At the location of the L4/5 spinal cord, we inserted a polyethylene-10 catheter into the subarachnoid space. An intrathecal catheter was implanted in the lumbar enlargement (close to the L4–5 segments) according to the method of Wu et al. (2004). A sudden movement of the tail or the hind limb indicated dura penetration. Following catheter implantation, the animals underwent 7 days of recovery prior to SNL or sham surgery. The rats were divided into six groups: naïve + scramble (miR-30b inhibitor N.C; 20 μM, 10 μl, GenePharma), naïve + miR-30b antagomir (a selective inhibitor of miR-30b), naïve + scramble (miR-30b agomir N.C), naïve + miR-30b agomir (a selective mimic of miR-30b), SNL + scramble (miR-30b agomir N.C) and SNL + miR-30b agomir. Beginning 1 day after naïve or 10 days after SNL surgery, continuous intrathecal infusion was delivered once a day for 4 days, from day 1 to 3 for naïve rats and from day 10 to 13 for SNL rats. Rats with neurological deficits were excluded from the experiment. The location of the intrathecal catheter was validated after the experiments (Guan et al., 2008; Xu et al., 2015).

### Behavioral Tests

#### Mechanical Paw Withdrawal Threshold

The latency of paw withdrawal response to mechanical stimulus was determined using the up–down method, following a previously described procedure (Malmquist et al., 2012). Mechanical paw withdrawal thresholds (PWTs) were measured on days 0, 3, 7, 14, and 21 for the evaluation of SNL model or on days 0, 3, 7, 10, 11, 12, 13, and 14 during continuous miR-30b agomir injection following SNL surgery. They were measured on days 0, 1, 2, 3, and 4 during continuous miR-30b antagomir injection in naïve rats, always between 8 and 10 AM in the morning. We placed each animal in a separate Plexiglas chamber on a raised wire screen, then we used Von Frey hairs (North Coast Medical Inc., Gilroy, CA, USA) in log increments of force (0.407, 0.692, 1.202, 2.041, 3.63, 5.495, 8.511, 15.14, and 26.0 g) to stimulate the plantar surface of the bilateral hind paws, beginning with the 2.041 g Von Frey hair. If the animal exhibited a positive reaction, the nearest smaller von Frey hair was applied; if a negative reaction was observed, the nearest larger von Frey hair was applied. The test was considered finished if one of two conditions were met: a negative reaction was acquired for the largest force (26.0 g) or three stimuli were performed after the first positive response. A formula provided by Dixon (Zang et al., 2010) was applied to convert the patterns of positive and negative reactions into a 50% threshold value (Coggeshall et al., 1997).

#### Thermal Paw Withdrawal Latency

The sample sizes and time points for the thermal tests were the same as those for the mechanical tests. The thermal paw withdrawal latency (PWL) was measured in the same manner as described by Kim and Chung (Coggeshall et al., 1997; Hargreaves et al., 1988; Malmquist et al., 2012). In Plexiglas chambers on a glass plate that could be heated through a hole in the light box by aiming a light beam, radiant heat was delivered to each hind paw through the glass plate, stimulating the middle of the plantar surface (UGO BASILE S.R.L., ITALY). When the rat lifted its foot, the beam of light was turned off. The time between the start of the beam of light and the lifting of the foot was considered as the PWL. Each test was repeated three times at 5 min intervals for each hind paw. A shut-off time of 15 s was applied to avoid any tissue injury.

### Luciferase Assay

A dual luciferase reporter assay was performed as outlined for a previous procedure (Huang et al., 2016). The pmirGLO dual-luciferase vector (pmirGLO vector), which contained both the firefly luciferase gene and the renilla luciferase gene, was purchased from Promega (Madison, WI, USA). SCN3A 3′UTR, including the predicted binding sites of miR-30b, was inserted into the 3′UTR region downstream of the firefly luciferase gene of the pmirGLO vector (pmir-GLO-UTR). A site-directed gene mutagenesis kit (GenePharma, Shanghai, China) was used to construct the mutant type of the miR-30b binding site vector (pmirGLO-mUTR) according to the protocol provided by the manufacturer. PC12 cells were cultivated in high glucose Dulbecco’s modified Eagle’s medium (Solarbio, Hyclone), which contained 5% fetal bovine serum (FBS, Gibco), 5% horse serum (Gibco), and 1% antibiotics (Gibco). The cells were incubated in a humidified incubator with 5% CO_2_ at 37°C. When the PC12 cells had a confluency of 70–80%, the reporter plasmids were determined to be fit to be transfected. After cultivation for 24 h, co-transfection of miRNA mimics (miR-30b agomir; GenePharma, Shanghai, China) at different doses of 10, 50, and 100 pM (50 nmol/L) with wild-type reporter vectors (0.5 μg/mL) was performed with Invitrogen lipofectamine 2000 (Invitrogen, Carlsbad, CA, USA). Then, co-transfection of other miRNAs with wild-type and mutant-type reporter vectors was conducted without serum medium or antibody as per the manufacturer’s instructions. After 6 h, we replaced the medium with a high glucose medium containing 1% antibiotics and 5% FBS. After another 48 h of culture, we used 1 × passive lysis buffer to lyse the transfected cells, and 20 μL supernatant was achieved for luciferase activity using the Dual-Luciferase Reporter Assay System (Promega). The ratio of firefly activity to renilla activity was recognized as relative reporter activity. Experiments were performed in triplicate and repeated three times.

### Cell Culture and Transfection

Culture and transfection of primary DRG neurons were carried out as described elsewhere (Zhao et al., 2013). Three-week-old rats were euthanized with isoflurane. All DRG neurons were collected in cold Neurobasal Medium (Gibco/ThermoFisher Scientific) with 10% FBS (JRScientific, Woodland, CA, USA), 100 μg/mL streptomycin, and 100 units/mL penicillin (Quality Biological, Gaithersburg, MD, USA). They were then treated with enzyme solution (1 mg/mL collagenase type I, 5 mg/mL dispase, in Hank’s balanced salt solution, excluding Mg^2+^ and Ca^2+^ [Gibco/Thermo Fisher Scientific]). The isolated cells were resuspended in mixed Neurobasal Medium and plated in a six-well plate coated with 50 μg/mL poly-D-lysine purchased from Sigma (St. Louis, MO, USA) with a seeding density of 10^5^ DRG neurons/mL. The cells were incubated at 37°C, 95% O_2_, and 5% CO_2_. One day later, 2 μL TNF-α (100 ng/mL, Peprotech) was added to each 2 mL well 30 min before the small miRNAs (GenePharma, Shanghai, China) were added. 100 μL Neurobasal Medium was used to dilute 5 μL (20 μM) miR-30b agomir/antagomir or 5 μL negative control (20 μM) for 5 min. One hundred microlitres Neurobasal Medium was simultaneously used to dilute 2 μL Lipofectamine 2000 (Invitrogen, Carlsbad, CA) for 5 min, then the two solutions were mixed. After 25 min, the mixture was placed into each 2 mL well and 800 Neurobasal Medium was added. The cells were collected 48 h later for PCR and western-blot examinations.

### Quantitative Reverse Transcription Polymerase Chain Reaction

For the quantitative real-time reverse transcription polymerase chain reaction (RT-PCR), two unilateral DRG neurons or 100 mg spinal cord (from the left side of one animal) were pooled to obtain sufficient RNA. Total RNA was extracted using Trizol reagent (Invitrogen), treated using DNase I (New England Biolabs, Ipswich, MA, USA), and reverse transcribed with the RevertAid First Strand cDNA Synthesis Kit (Thermo). The miRNA was reverse transcribed with an miRcute miRNA First Strand cDNA Synthesis Kit (TIANGEN). A template (2 μL) was used for amplification by real-time PCR with random hexamers, oligo (dT) primers, or specific RT primers, as shown in **Table 1**. GAPDH and u6 were taken as internal controls for normalization. Each sample was run in triplicate in a 20 μL volume for reaction with 250 nM forward and reverse primers, 10 μL Thermo Scientific Maxima SYBR Green qPCR Master Mix (2×; Thermo Scientific Maxima SYBR Green qPCR Master Mix, Rox solution provided), and 20 ng total cDNA. For miRNA quantitative real-time RT-PCR, a miRcute miRNA qPCR Detection Kit (SYBR Green, TIANGEN, Beijing, China) was used. Reactions were implemented in a 7500 Fast Real-Time PCR Detection System (Applied Biosystems, USA). The ratios of the SNL-operated mRNA level to the sham-operated mRNA level were calculated using the ÄCt method (2^-ÄÄCt^). All SCN3A data were normalized to GAPDH, and all miR-30b data were normalized to u6, which was confirmed to be stable (Zhao et al., 2013; Huang et al., 2016).

**Table 1:** Primer sets used for qRT-PCR for rat samples.

Gene name	Primer sequence
SCN3A	5′-TATCCGTGTCAACTGGAC-3′ 5′-ACTTGTGGACTTAGCAAC-3′
GAPDH	5′-TCG GTG TGA ACG GAT TTG GC-3′ 5′-CCT TCA GGT GAG CCC CAG C-3′
U6	5′-GCT TCG GCA GCA CAT ATA CTA A-3′ 5′-CGA ATT TGC GTG TCA TCC TT-3′
miR-30b	5′-CCAGCAACTGTAAACATCCTACAC-3′ 5′-TATGGTTTTGACGACTGTGTGAT-3′

### Western Blot

To ensure a sufficient amount of protein, two unilateral rat DRG neurons were pooled together and a section of ipsilateral Lumbar enlargement was prepared. Based on established protocol (Zhao et al., 2015), tissues were homogenized in a chilled lysis buffer (10 mM Tris, 1 mM phenylmethylsulfonyl fluoride, 5 mM MgCl_2_, 5 mM EGTA, 1 mM EDTA, 1 mM DTT, 40 μM leupeptin, 250 mM sucrose). After centrifugation at 4°C for 15 min at 1,000 × *g*, the supernatant was collected to analyze cytosolic proteins and the pellet was collected to analyze nuclear proteins. The contents of the proteins in the samples were measured using the Bio-Rad protein assay (Bio-Rad) and were then heated at 99°C for 5 min. Samples of 30 μg total protein were separated by 6% SDS-polyacrylamide gel electrophoresis and electrophoretically transferred onto a polyvinylidene difluoride membrane. After the membranes were blocked with 3% BSA (Solarbio, Beijing, China) in Tris-buffered saline containing 0.1% Tween-20 for 3 h, rabbit anti-Nav1.3 (1:300, Borson) and rabbit anti-β-actin (1:1000, Zhongshan Jinqiao, China) primary antibodies would be used. The proteins were detected using horseradish peroxidase-conjugated anti-rabbit secondary antibody (1:1000, Jackson) and visualized using Western peroxide reagent and luminol/enhancer reagent (Clarity Western ECL Substrate, Bio-Rad); the intensity exposure using t using FluorChem E (Alphalmager proteinsimple, San Jose, CA, USA). The intensity of the blots was quantified via densitometry using Image Lab software (Bio-Rad). All cytosol protein bands were normalized to β-actin.

### Immunofluorescence

Rats were perfused with 4% paraformaldehyde after they were anesthetized with isoflurane for the preparation of double-labeled immunohistochemistry, as described previously (Xu et al., 2013; Wang et al., 2013). L4 and L5 DRG neurons were removed, post-fixed, and dehydrated before frozen sectioning at 16 μm. After the sections were blocked for 1–2 h in 0.01 M PBS containing 10% goat serum and 0.3% Triton X-100 at room temperature, they were incubated with the following primary antibodies over one or two nights at 4°C. The antibodies and reagents included rabbit anti-Nav1.3 (1:800, Abcam), mouse anti-NF200 (1:200, Abcam), biotinylated IB4 (1:100, Sigma), mouse anti-CGRP (1:50, Abcam), mouse anti-Gelsolin (GS; 1:200, R&D), rabbit anti-NF200 (1:200, Abcam), rabbit anti-CGRP (1:50, Abcam), and rabbit anti-Gelsolin (GS; 1:200, R&D). The sections were then incubated with either goat anti-rabbit antibody conjugated to Cy3 (1:200, Jackson Immunity Research, West Grove, PA, USA) or goat anti-mouse antibody conjugated to Cy2 (1:200, Jackson Immunity Research) for 2 h in the incubator at 37°C. All immunofluorescence-stained images were examined using a Leica DMI4000 fluorescence microscope and captured with a DFC365FX camera (Leica, Germany). Double-stained neurons were quantified manually or by using NIH Image J Software.

### *In Situ* Hybridization

The L5 DRGs of animals were prepared for measurements and perfused intracardially with 0.01 M PBS followed by 4% cold buffered paraformaldehyde. They were sectioned at 16 μm and frozen after they were post-fixed in 4% paraformaldehyde for 30 min and dehydrated overnight in 30% sucrose at 4°C.

The rat miR-30b *in situ* hybridization Assay kit we used in this experiment was purchased from Boster Bio-Tech (Wuhan, China). The special probe sequence for miR-30b was as follows: 5′—AGCTG AGTGT AGGAT GTTTA CA—3′. We performed the experiment as per the protocol provided by the manufacturer. In brief, we first mixed 30% H_2_O_2_ with pure methanol in a 1:50 ratio before dropping it to each section at room temperature for 30 min, then washed three times with distilled water. 3% citric acid was added to sections to expose the mRNA (two drops pepsase in 1 mL 3% citric acid) for 2 min at room temperature. We then washed the section with PBS three times at intervals of 5 min, using distilled water during the third wash. Later, we post-fixed it with 1% paraformaldehyde 0.1 M PBS at room temperature for 10 min and washed again. We added 20 μL preliminary hybrid liquid to each section and incubated them for 2–4 h at 37°C with one humidified box of 20 mL 20% glycerin for pre-hybridization. Afterward, 20 μL hybrid liquid would be applied to them overnight in the incubator for hybridization. We then washed each section with 2 × SSC two times at an interval of 5 min and with 0.5 × SSC and 0.2 × SSC, one time each, for 15 min post-hybridization. After they were blocked for 30 min at 37°C, we incubated them with anti-rat biotin digoxin at 37°C for 60 min or at room temperature for 120 min. Finally, SABC-FITC/CY3 was used for fluorescent coloring, and the mRNA cell cytoplasm was colored green/red for visibility under a fluorescence microscope.

### Statistical Analysis

The data are presented as means ± SEM. For comparisons between two groups, the *P* value was evaluated and calculated using a two-tailed paired *t*-test. When there were multiple factors involved, a two-way analysis of variance (ANOVA) was used; multiple groups were compared using a one-way or two-way ANOVA. Values of *P* < 0.05 were considered statistically significant.

## Results

### MiR-30b Directly Targets SCN3A by Binding with the 3′UTR of SCN3A in PC12 Cells

**FIGURE 1:**
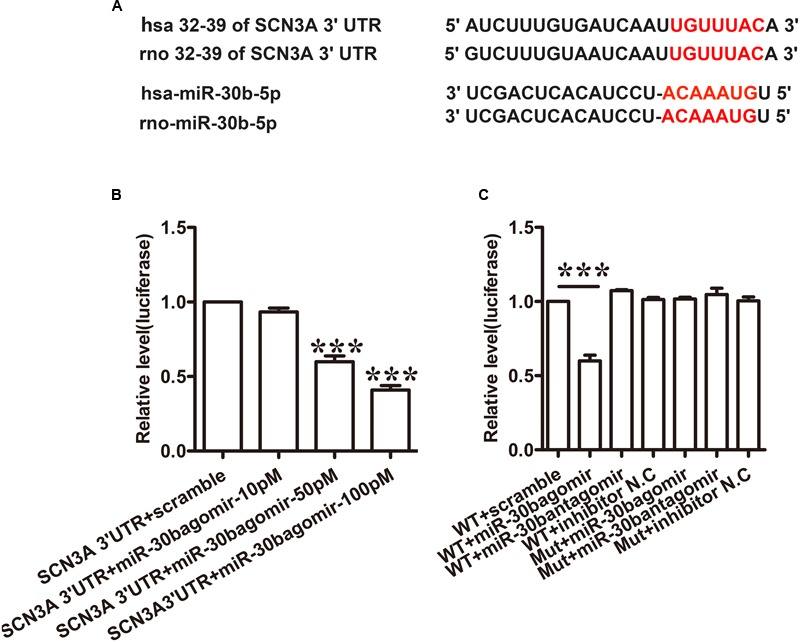
**miR-30b directly targets SCN3A 3′UTR. (A)** The matched seed region between miR-30b and SCN 3′UTR predicted by Target Scan is in red. **(B)** MiR-30b agomir decreased relative luciferase activity in a dose-dependent manner with wild-type (WT) SCN3A 3′UTR plasmid vector in PC12 cells, ^∗∗∗^*P* < 0.0001 vs. scramble. One-way ANOVA, *n* = 3. **(C)** Transfection of miR-30b agomir with WT SCN3A 3′UTR reduced relative luciferase activity, but no change in luciferase activity was detected in the scramble or mutant SCN3A 3′UTR group, ^∗∗∗^*P* < 0.0001 vs. WT+ scramble, *n* = 3. Data are shown as means ± SEM.

Tetrodotoxin-sensitive VGSC Nav1.3 was encoded by SCN3A. We discovered that SCN3A was the primary target of miR-30b using Target Scan software. The matched seed sequences between miR-30b-5p and SCN3A 3′UTR were highly conserved between human and rats as shown in **Figure 1A**. The Target Scan software demonstrated that the seed sequence of the miR-30b position (2–8) was paired with SCN3A 3′UTR from 32–39 bps in both humans and rats.

To verify whether miR-30b targets SCN3A 3′UTR, a dual luciferase reporter vector containing the sequence of SCN3A 3′UTR was designed (pmirGLO SCN3A 3′UTR). Transfecting the wild-type plasmid vector with three different doses of miR-30b agomir (10, 50, and 100 pM) into PC12 cells with Lipofectamine 2000, miR-30b agomir reduced relative luciferase activity in a dose-dependent manner (**Figure 1B**, ^∗∗∗^*P* < 0.0001). However, the luciferase activities of miR-30b antagomir and scrambled miRNAs were unchanged (**Figure 1C**, *P* > 0.05), indicating that the inhibition of miR-30b agomir was sequence specific. To further prove the specificity of SCN3A 3′UTR, we transfected mutant 3′UTR plasmid with miR-30b agomir into PC12 cells. As expected, miR-30b had no effect on luciferase activity (**Figure 1C**).

### MiR-30bagomir Transfection Is Essential to Inhibit the Expression of Nav1.3 mRNA in Primary DRG Neurons

**FIGURE 2:**
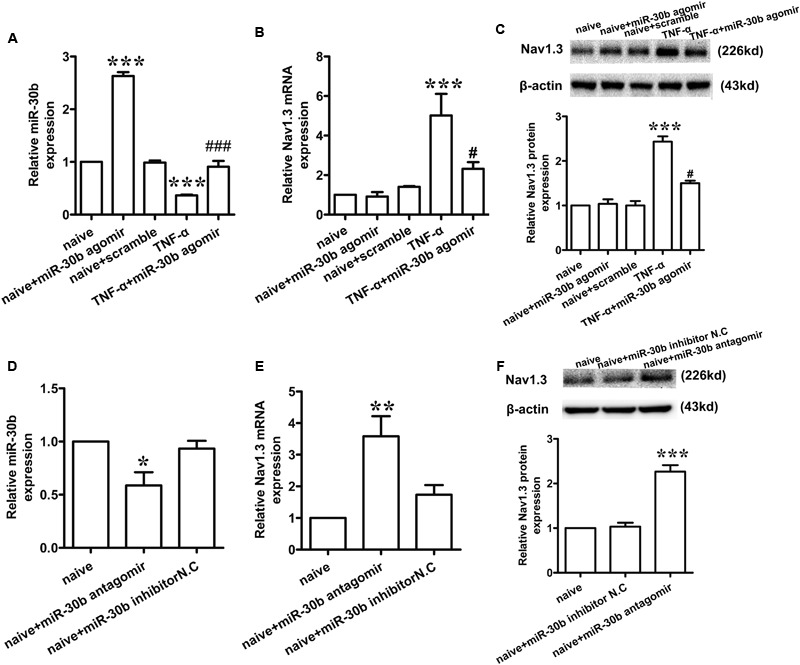
**miR-30b regulated the expression of Nav1.3 in primary cultured DRG neurons. (A–C)** The relative expression of miR-30b **(A)** and Nav1.3 mRNA **(B)** and protein **(C)** treated with TNF-α and miR-30b agomir/scramble in primary cultured DRG neurons, ^∗∗∗^*P* = 0.0007, ^∗∗∗^*P* = 0.0010 vs. naïve, ^###^*P* < 0.0001 vs. TNF-α; ^∗∗∗^*P* = 0.0003 vs. naïve, ^#^*P* = 0.042 vs. TNF-α; ^∗∗∗^*P* < 0.0001 vs. naïve, ^#^*P* = 0.0162 vs. TNF-α. One-way ANOVA, *n* = 3 rats. **(D–F)** The relative expression of miR-30b **(D)** and Nav1.3 mRNA **(E)** and protein **(F)** treated with miR-30b antagomir in primary cultured DRG neurons, ^∗^*P* = 0.0140, ^∗∗^*P* = 0.0043, ^∗∗∗^*P* = 0.0001 vs. naïve, one-way ANOVA, *n* = 3 rats. Data are shown as means ± SEM.

To determine whether miR-30b may regulate the expression of Nav1.3, we used TNF-α (2 μL, 100 ng/mL) to stimulate the primary DRG neurons. 30 min later, we transfected miR-30b agomir. The levels of miR-30b and SCN3A mRNA were measured by qRT-PCR and the changes in Nav1.3 protein expression were determined by western-blot. Compared to the naïve non-transfected group, TNF-α stimulation induced a significant increase in Nav1.3 at mRNA (**Figure 2B**, ^∗∗∗^*P* = 0.0003) and protein level (**Figure 2C**, ^∗∗∗^*P* < 0.0001), while a reduction of miR-30b was observed (**Figure 2A**, ^∗∗∗^*P* = 0.0007). However, miR-30b overexpression, by transfecting miR-30b agomir, reversed the up-regulation of SCN3A (**Figure 2B**, ^#^*P* = 0.042) and Nav1.3 (**Figure 2C**, ^#^*P* = 0.0162) and attenuated the down-regulation of miR-30b (**Figure 2A**, ^###^*P* < 0.0001). Moreover, miR-30b agomir transfection increased the expression of miR-30b (**Figure 2A**, ^∗∗∗^*P* = 0.0010) but did not influence SCN3A (**Figure 2B**, *P* = 0.73) or Nav1.3 (**Figure 2C**, *P* = 0.75) in untreated DRG cells. In addition, we found that miR-30b antagomir transfection up-regulated Nav1.3 (**Figures 2E,F**), while it down-regulated miR-30b (**Figure 2D**, ^∗^*P* = 0.014). On the other hand, the role of endogenous miR-30b in regulating Nav1.3 expression was identified in primary DRG neurons. Taken together, we demonstrated that miR-30b suppressed the expression of Nav1.3 mRNA by binding with SCN3A 3′UTR.

### Up-Regulation of Nav1.3 Is Inversely Correlated with Down-Regulation of miR-30b in SNL Rats

**FIGURE 3:**
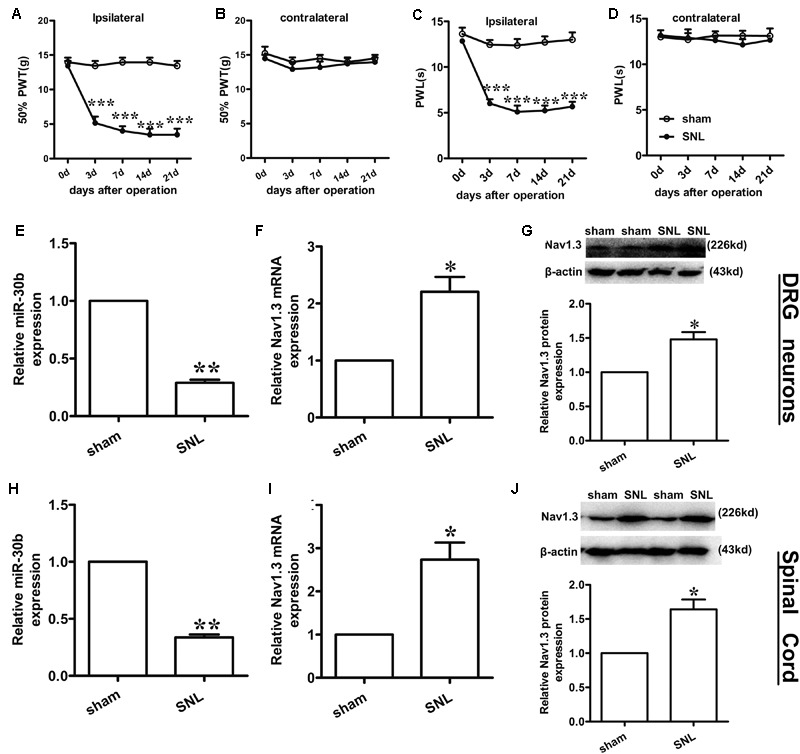
**Spinal nerve ligation-induced mechanical and thermal allodynia and the change in expression of Nav1.3 and miR-30b. (A,C)** Responses of the ipsilateral paw to mechanical and thermal stimuli, ^∗∗∗^*P* < 0.001 vs. sham, two-way ANOVA, *n* = 6 rats; **(B,D)** Responses of the contralateral paw to mechanical and thermal stimuli, *P* > 0.05 vs. sham, two-way ANOVA, *n* = 6 rats. **(E–G)** The decreased expression of miR-30b **(E)** and increased expression of Nav1.3 mRNA **(F)** and protein **(G)** in DRG neurons of SNL rats, ^∗∗^*P* = 0.0013, ^∗^*P* = 0.043, ^∗^*P* = 0.045 vs. sham, two-tailed paired *t*-test, *n* = 3 rats. **(H–J)** The decreased expression of miR-30b **(H)** and increased expression of Nav1.3 mRNA **(I)** and protein **(J)** in spinal cord of SNL rats, ^∗∗^*P* = 0.0015, ^∗^*P* = 0.0488, ^∗^*P* = 0.0471 vs. sham, two-tailed paired *t*-test, *n* = 3 rats. Data are shown as means ± SEM.

Compared to baseline pre-injury values observed at day 0 (50% PWTs and (10–15) g PWLs and (10–15) s), L5 SNL induced a conspicuous reduction in PWTs (**Figure 3A**, ^∗∗∗^*P* < 0.0001) and PWLs (**Figure 3C**, ^∗∗∗^*P* < 0.0001) of the ipsilateral hindpaw of the injured side from day 3 to 21 post-SNL, but did not change the basal contralateral PWTs (**Figure 3B**, *P* = 0.96) and PWLs (**Figure 3D**, *P* = 0.82) during the observation period. By comparison, no changes in the mechanical and thermal thresholds for paw withdrawal were observed in the sham-operated group.

To determine the expression of miR-30b and Nav1.3 in DRG neurons and the spinal cord of SNL rats, we performed qRT-PCR and western blot analysis (tissues were acquired at day 14 post-SNL surgery). Compared to sham-operated rats, SNL caused an obvious down regulation of miR-30b expression (**Figure 3E**, ^∗∗^*P* = 0.0013) and up-regulation of Nav1.3 mRNA expression in DRG neurons (**Figure 3F**, ^∗^*P* = 0.043) as well as in the spinal cord (**Figures 3H,I**). Western blot results showed that Nav1.3 protein strongly increased after nerve injury (**Figures 3G,J**), consistent with the data from the behavioral test. As a consequence, the increased expression of Nav1.3 mRNA and protein and the decreased expression of miR-30b in the DRG and spinal cord of SNL rats confirmed the potential ability of miR-30b to alleviate SNL-induced neuropathic pain.

### MiR-30b Is Co-Localized with Nav1.3 in DRG Neurons

**FIGURE 4:**
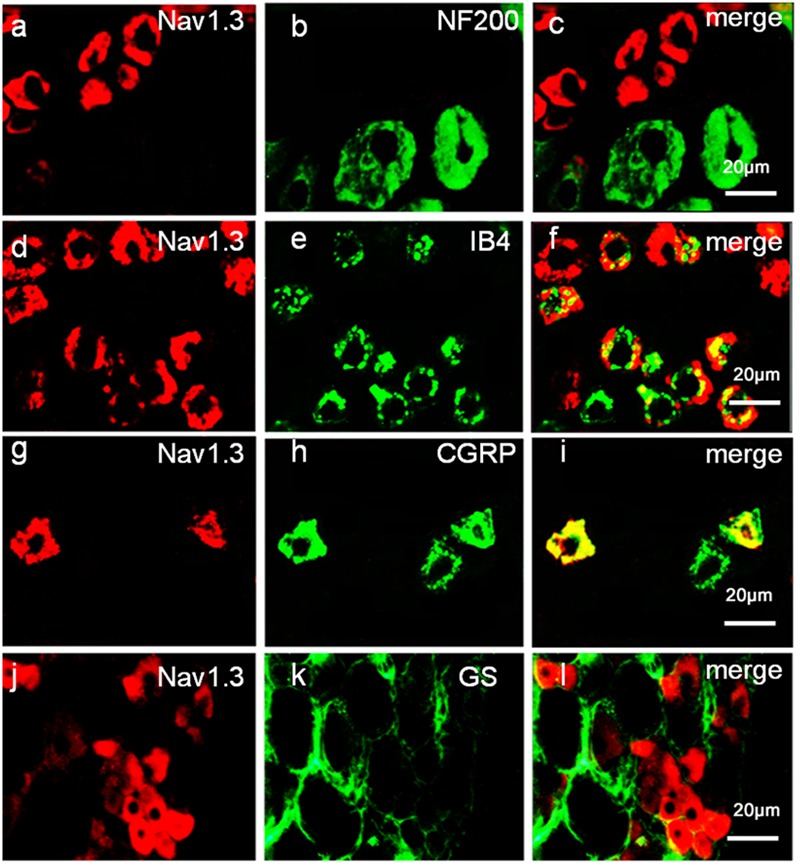
**Expression distribution of Nav1.3 protein in DRG neurons of SNL rats.** Nav1.3 was double-stained with IB4 **(d–f)** and CGRP **(g–i)**; but Nav1.3 was not found to stain with NF200 **(a–c)** and GS **(j–l)**. Immunofluorescence staining of IB4 **(d–f)** and CGRP **(g–i)** show that Nav1.3 was mainly co-localized with nociceptive neuronal marker, *n* = 3 rats. Scale bar: 20 μm.

**FIGURE 5:**
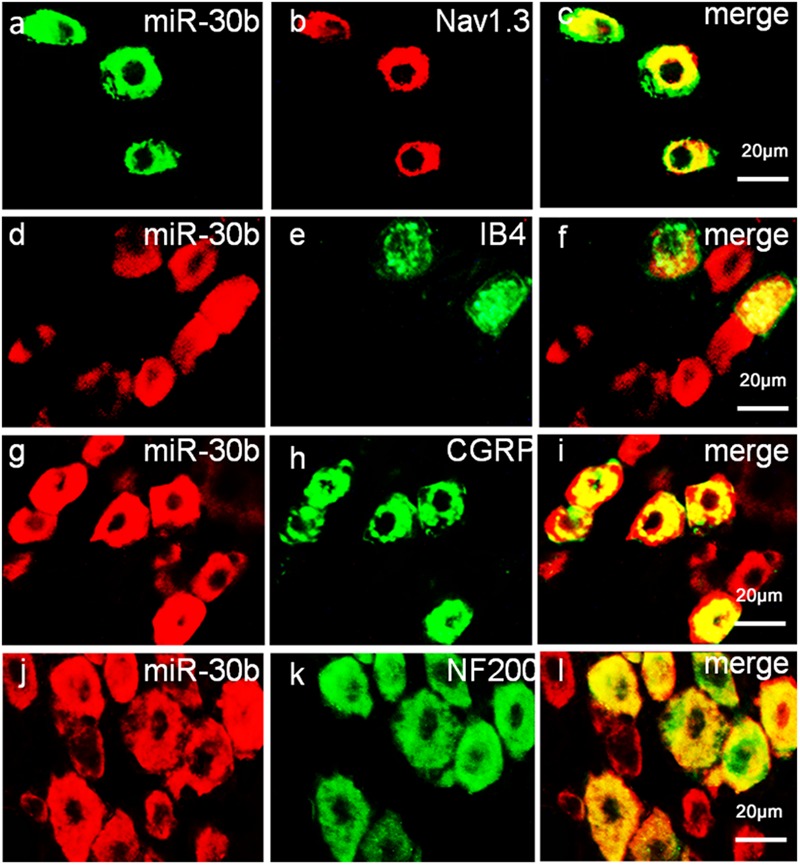
**Expression distribution of miR-30b and co-localization with Nav1.3 in DRG neurons of naïve rats.** *In situ* hybridization of miR-30b and immunofluorescence staining of NF200 **(a–c)**, IB4 **(d–f)**, CGRP **(g–i)**, and NF200 **(j–l)** showed that miR-30b was co-localized with nociceptive neuronal and non-nociceptive neurons marker and miR-30b was co-localized with Nav1.3 **(a–c)**, *n* = 3 rats. Scale bars: 20 μm.

To define the localization of Nav1.3 and miR-30b, double-labeled immunofluorescence and *in situ* hybridization were performed in DRG neurons. As shown in **Figure 4**, we stained Nav1.3 with NF-200 (a–c), a marker of large myelinated non-nociceptive neurons, CGRP (g–i), a marker for small nociceptive peptidergic neurons, IB4 (d–f), a marker for a fraction of small, non-myelinated nociceptive neurons and GS (j–l), a marker for glial cells. Results showed that the Nav1.3 signal was mainly double-labeled with IB4 and CGRP (f, i) while it was not found to localize with NF-200 and GS (c, l). In **Figure 5**, *in situ* hybridization results expressed that miR-30b was double-labeled with NF200, IB4, and CGRP (f, i, l). Importantly, the cells containing miR-30b express Nav1.3 in DRG neurons (a–c), indicating a potential interaction between miR-30b and Nav1.3.

### Intrathecal miR-30b Agomir Inhibits the Expression of Nav1.3 in DRG and Spinal Cord and Attenuates Neuropathic Pain in SNL Rats

**FIGURE 6:**
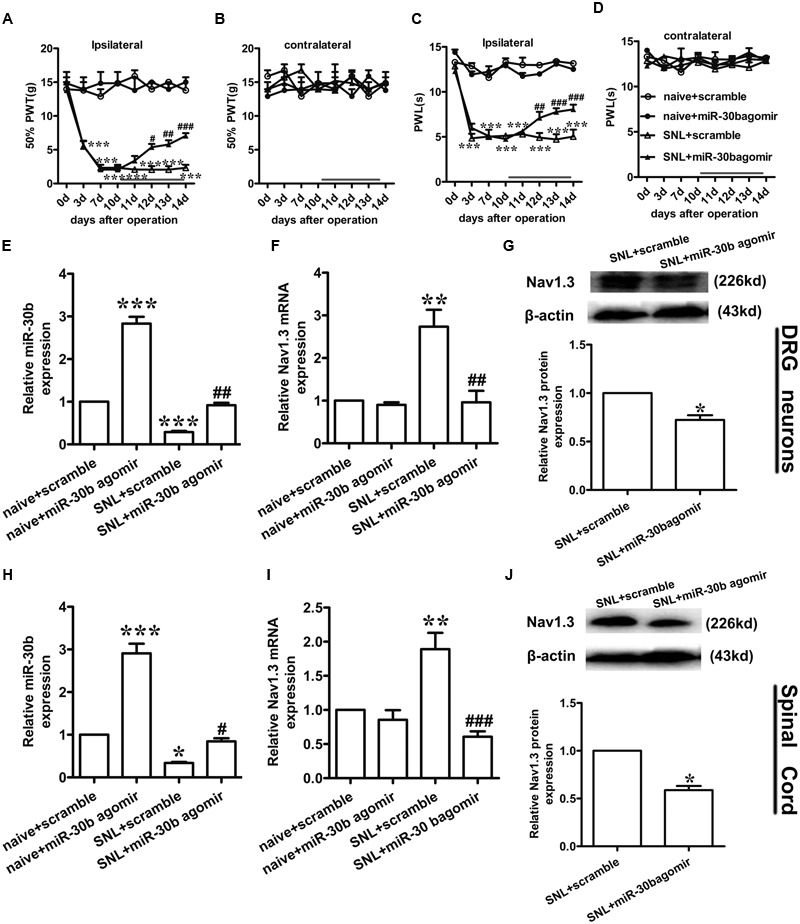
**miR-30b agomir down regulated Nav1.3 and alleviated neuropathic pain. (A,C)** Ipsilateral paw withdrawal to mechanical and thermal thresholds, ^∗∗∗^*P* < 0.001 vs. naïve + scramble; ^#^*P* = 0.025, ^##^*P* = 0.0062, ^###^*P* = 0.00048; ^#^*P* = 0.032, ^###^*P* = 0.00053, ^###^*P* = 0.00025 vs. SNL + scramble, two-way ANOVA, *n* = 6 rats. **(B,D)** Contralateral paw withdrawal to mechanical and thermal thresholds, *P* > 0.05 vs. naïve + scramble; *P* > 0.05 vs. SNL + scramble, two-way ANOVA, *n* = 6 rats. **(E,F)** The relative expression of miR-30b **(E)** and SCN3A **(F)** with intrathecal miR-30b agomir in DRG neurons of SNL rats was determined by qRT-PCR, ^∗∗^*P* < 0.01, ^∗∗∗^*P* < 0.001 vs. naïve + scramble; ^##^*P* = 0.0034, ^##^*P* = 0.0018 vs. SNL + scramble, one-way ANOVA, *n* = 3 rats. **(G)** Nav1.3 protein expression in DRG neurons of SNL rats at 14 days injected with intrathecal miR-30b agomir, ^∗^*P* = 0.0303 vs. SNL + scramble, two-tailed paired *t*-test, *n* = 3 rats. **(H,I)** The relative expression of miR-30b **(H)** and SCN3A **(I)** with intrathecal miR-30b agomir in spinal cord of SNL rats, ^∗^*P* < 0.05, ^∗∗^*P* < 0.01 ^∗∗∗^*P* < 0.001 vs. naïve + scramble; ^#^*P* = 0.0366, ^###^*P* = 0.00087 vs. SNL + scramble, one-way ANOVA, *n* = 3 rats. **(J)** Nav1.3 protein expression in spinal cord of SNL rats at 14 days injected with intrathecal miR-30b agomir, ^∗^*P* = 0.0110 vs. SNL + scramble, two-tailed paired *t*-test, *n* = 3 rats. Data are shown as means ± SEM.

To assess the exact impact of miR-30b on neuropathic pain, we delivered miR-30b agomir to SNL rats for 4 days following day 10 with intrathecal injection, and 50% PWTs and PWLs were tested. At day 10 after SNL, neuropathic pain was established (^∗∗∗^*P* < 0.0001). From day 2 following drug administration, the mechanical allodynia (**Figure 6A**) and thermal hyperalgesia (**Figure 6C**) caused by SNL were attenuated by intrathecal injection with miR-30b agomir, not scrambled miRNA, but the thresholds of the contralateral hind paw were unchanged (**Figures 6B,D**, *P* > 0.05). MiR-30b agomir did not affect the baseline of PWTs (**Figure 6A**) and PWLs (**Figure 6C**) in naïve rats.

**FIGURE 7:**
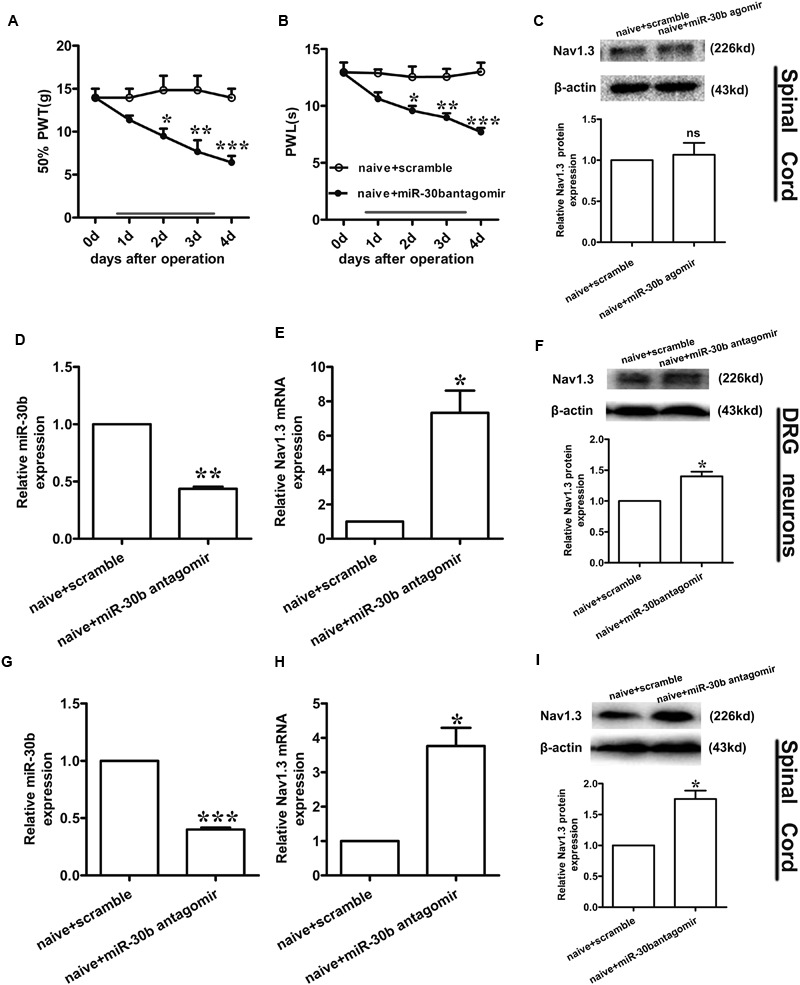
**Nav1.3 was upregulated by miR-30b antagomir with pain behaviors. (A,B)** Responses to mechanical and thermal stimulus, ^∗^*P* < 0.05, ^∗∗^*P* < 0.01, ^∗∗∗^*P* < 0.001 vs. naïve + scramble, two-way ANOVA, *n* = 6 rats. **(C)** Nav1.3 protein expression in spinal cord of rats injected with miR-30b agomir, *P* = 0.6914 vs. naïve + scramble, two-tailed paired *t*-test, *n* = 3 rats. **(D,E)** The relative mRNA expression of miR-30b **(D)** and SCN3A **(E)** with intrathecal miR-30b antagomir in DRG neurons of naïve rats, ^∗^*P* = 0.0394, ^∗∗^*P* = 0.0011 vs. naïve + scramble, one-way ANOVA, *n* = 3 rats. **(F)** Nav1.3 protein expression in DRG neurons of naïve rats at 4 days injected with intrathecal miR-30b antagomir, ^∗^*P* = 0.0341 vs. naïve + scramble, two-tailed paired *t*-test, *n* = 3 rats. **(G,H)** The relative mRNA expression of miR-30b **(G)** and SCN3A **(H)** with intrathecal miR-30b antagomir in spinal cord of naïve rats, ^∗^*P* = 0.0344, ^∗∗∗^*P* = 0.0009 vs. naïve + scramble, one-way ANOVA, *n* = 3 rats. **(I)** Nav1.3 protein expression in spinal cord of naïve rats at 4 days injected with intrathecal miR-30b antagomir, ^∗^*P* = 0.0309 vs. naïve + scramble, two-tailed paired *t*-test, *n* = 3 rats. Data are shown as means ± SEM.

To test whether miR-30b agomir could repress the expression of Nav1.3, we measured the expression of miR-30b and Nav1.3 by qPCR and western blot (tissues were acquired at day 14 post-SNL surgeon). MiR-30b agomir reversed the upregulation of SCN3A (**Figures 6F,I**) and downregulation of miR-30b (**Figures 6E,H**) in SNL rats. Meanwhile, in naïve rats, it increased miR-30b levels (**Figures 6E,H**) but had no influence on the expression of SCN3A (*P* > 0.05). In western-blot data, the upregulation of Nav1.3 protein was effectively inhibited by miR-30b agomir in DRG neurons (**Figure 6G**, ^∗^*P* = 0.0303) and spinal cord (**Figure 6J**, ^∗^*P* = 0.0110) in SNL rats. In accordance with the mRNA level of Nav1.3 in naïve rats, the protein expression of Nav1.3 had no significant change between scramble and miR-30b agomir injected in spinal cord (**Figure 7C**, *P* = 0.6914). These results confirm that miR-30b overexpression reverses the upward tendency of Nav1.3 in SNL rats at the level of mRNA and protein, leading to a partial easement of pain.

### Intrathecal miR-30b Antagomir Increases the Expression of Nav1.3 in Naïve Rats

To further explore the regulation of Nav1.3 by miR-30b, we down regulated miR-30b by intrathecal injection with miR-30b antagomir in naïve rats. We applied miR-30b antagomir to naïve rats for 4 days and determined their sensitivity to mechanical and thermal stimulus. We found that the threshold values for mechanical and thermal stimulus were significantly lower during miR-30b antagomir delivery than those of naïve rats injected with scrambled miRNAs (**Figures 7A,B**), demonstrating that miR-30b antagomir produced pain behaviors in naïve rats. Furthermore, ipsilateral L4-L5 DRGs and spinal cord were acquired at day 4 in order to assess expression of Nav1.3 at the mRNA and protein levels. Down regulation of miR-30b (**Figures 7D,G**) induced increases in Nav1.3 mRNA in DRG neurons (**Figure 7E**, ^∗∗^*P* = 0.0011) and in spinal cord (**Figure 7H**, ^∗^*P* = 0.0344), as well as in protein expression (**Figures 7F,I**).

## Discussion

Nav1.3, an isform of the tetrodotoxin-sensitive (TTX-S) VGSC, was capable of producing sodium ion currents with rapid repriming dynamics that can facilitate neuronal hyperexcitability, enhanced repetitive firing characteristics and ectopic discharge in injured neurons (Waxman and Hains, 2006). Nav1.3 has been reported to be upregulated in different pain states, such as STZ-induced pain (Tan et al., 2015), nerve transection and chronic constriction injury models (CCI) of neuropathic pain with pain behaviors (Hains et al., 2005; Samad et al., 2013; Chen et al., 2014). Downregulation of Nav1.3 mRNA and protein through intrathecal administration of AS ODNs can decrease neuronal hyperexcitability and alleviate mechanical allodynia and thermal hyperalgesia following Spinal Cord Injury (SCI) (Hains et al., 2003). Samad et al. (2013) showed that knockdown of Nav1.3 was able to relieve neuropathic pain. Accordingly, in the present study, the expression of Nav1.3 mRNA and protein increased in SNL rats, not only in DRG neurons (**Figures 3F,G**; Hains et al., 2005; Samad et al., 2013; Chen et al., 2014) but also in the spinal cord (**Figures 3I,J**). In comparison to Hains et al. (2003), which stated that Nav1.3 expression was increased in the spinal cord following SCI, the increased Nav1.3 expression we observed in the spinal cord in SNL rats was likely a consequence of the increased expression in DRG neurons that end up in superficial layers of the spinal cord. But we preferred another explanation, as it appeared that peripheral nerve injury induced central hyperalgesia through some signaling pathways or inflammatory cytokines, leading to the up-regulation of Nav1.3 in spinal dorsal horn neurons. However, the underlying mechanism was still unknown, which would require further experiments to prove. Additionally, Nav1.3 was mostly double-labeled with IB4 and CGRP (**Figures 4F,I**), which are markers of C fibers that are essentially involved in nociceptive information transfer. These findings, together with our results, strongly suggested that Nav1.3 played a crucial role in neuropathic pain. Despite recent advances, understanding the transcriptional or translational regulatory mechanisms underlying the changes in expression and function of Nav1.3 remained a major challenge.

In recent years, non-coding RNAs have been extensively researched. NcRNAs participate in the regulation of numerous cellular processes, which might modulate disease onset, progression and prognosis. MiRNAs have widely existed *in vivo*, and were implicated in the post-transcription regulation of gene expression by repressing mRNA translation or inhibiting mRNA and protein degradation (Bartel, 2004; Lutz et al., 2014). There has been a focus on studies that have associated miRNAs with chronic neuropathic pain states. MiR-132 was upregulated in SNI rats (Leinders et al., 2016), while miR-182, miR-183, miR-96 decreased in SNL rats (Aldrich et al., 2009). In particular, miR-96 was also involved in CCI model (Chen et al., 2014). These findings provide us with information that we can use to identify major players in neuropathic pain mechanisms.

Using Target Scan software, miR-30b, miR-96, miR-183, and miR-132 were found to target SCN3A. In a recent study, miR-183 and miR-96 were observed a significant down-regulation with the increase of Nav1.3 expression in L5 DRG after SNL, which were abundant in DRG neurons (Aldrich et al., 2009; Lin et al., 2014), overexpression of miR-183 and miR-96 were capable to attenuate neuropathic pain by repressing Nav1.3. During the study, we focused on miR-30b and attempted to explore the potential role of miR-30b and SCN3A in SNL rats. Through Luciferase assay we verified that miR-30b negatively regulated SCN3A by combining with SCN3A 3′UTR (**Figure 1B**). As expected, the transfection of scrambled miRNA or mutant SCN3A 3′UTR was not able to change the Firefly/Rellia ratio significantly (**Figures 1B,C**, *P* > 0.05), indicating that miR-30b and the 3′UTR of SCN3A were specific. Moreover, immunofluorescence and *in situ* hybridization determined that miR-30b was co-localized with Nav1.3 in rat DRGs (**Figure 5**), providing evidence for the interaction between miR-30b and Nav1.3.

TNF-α that could increase VGSC mRNA quantity and the number of available channels in the plasma membrane was used to stimulate the primary DRG neurons. In accordance with Chen et al. (2015), Nav1.3 was increased at both the mRNA and protein levels at the stimulation of TNF-α. The increased expression of Nav1.3 (**Figures 2B,C**) proved that the enhanced excitability of neurons induced by TNF-α was mediated by the up-regulation of Nav1.3. Consistently (Zang et al., 2011), administration of rrTNF to primary DRG neurons induced Nav1.3 re-expression. Furthermore, increased Nav1.3 levels were inhibited by the transfection of miR-30b agomir (**Figure 2B**, ^#^*P* = 0.042; **Figure 2C**, ^#^*P* = 0.0162). Hence, miR-30b was validated to regulate Nav1.3 at transcription level. Meanwhile, transfecting miR-30b agomir did not alter the level of SCN3A in naïve DRG neurons (**Figure 2C**, *P* = 0.75; **Figure 7C**, *P* = 0.6914), which seemed to be ambivalent with the results we acquired from TNF-α treated group or from SNL rats, however, it did match with the characteristics of SCN3A, which was almost undetectable in adult neurons (Estacion et al., 2010), consequently, miR-30b agomir failed to induce changes in the expression of SCN3A in naïve rats.

Similar to our previous study (Shao et al., 2016), miR-30b was proved to ease neuropathic pain by regulating SCN9A after SNI. In the present study, we demonstrated that miR-30b alleviated pain by inhibiting SCN3A through the evaluation of behaviors and changes in molecular levels in SNL rats. The observed pain-related behaviors were consistently recovered (**Figures 3A,C**), meanwhile, the increased expression of Nav1.3 was found to reverse (**Figures 3F,G,I,J**) with intrathecal administration miR-30b agomir in SNL rats, which just verified that a single miRNA was able to act on multiple target genes. The finding that Nav1.3 and Nav1.7 were both regulated by miR-30b in neuropathic pain emphasized the importance role of miR-30b in different models of neuropathic pain, implying that miR-30b might be a practicable drug target for the treatment of neuropathic pain. Likewise, one gene was likely to be targeted by multiple miRNAs. SCN3A was not only targeted by miR-30b, but also controlled by miR-183 and miR-96 in SNL rat DRGs (Aldrich et al., 2009). These accumulated evidence revealed that miR-30b and SCN3A were crucial players in neuropathic pain, thus, illustrating the potential mechanism would provide a new direction and serviceable theoretical foundation for the clinical intervention of neuropathic pain.

Moreover, we found that intrathecal administration of miR-30b antagomir contributed to pain behaviors (**Figures 7A,B**) and the up-regulation of Nav1.3 (**Figures 7E,F**, DRG neurons; H,I, spinal cord) in naïve rats. In conformity to previous report (Leinders et al., 2016), intrathecal injection of miR-132-3p mimetic dose-dependently produced pain behavior in naïve rats, miR-132-3p was reported to up-regulated in neuropathic pain, which was in contrast to miR-30b. Even so, the effect of miR-30b antagomir was not unsustainable given the momentariness, along with miR-30b agomir, which have to be settled urgently.

There are some limitations in our study. Firstly, the upstream molecules of miR-30b remain uncertain. Secondly, miR-30b was involved in neuropathic pain by targeting several proteins, and we did not evaluate all of the possible targets of miR-30b. Thirdly, Nav1.3 was reported to take part in STZ-induced pain (Tan et al., 2015) and Nav1.7 was changed in inflammatory pain (Yeomans et al., 2005), but whether miR-30b participated in STZ-induced pain or inflammatory pain by targeting SCN3A or SCN9A confused us. Prominently, it is essential to supplement patch clamp recording in our follow-up work because of the contribution of VGSCs to physiological and pathophysiological electrical signaling (McCormack et al., 2013). However, it did address the fact that miR-30b directly regulated SCN3A and further demonstrated that miR-30b had a potential use for the therapy invention for the treatment of neuropathic pain.

## Conclusion

We found that miR-30b directly targeted SCN3A 3′UTR both *in vitro* and *in vivo*, and that miR-30b alleviated neuropathic pain by suppressing the expression of Nav1.3 in DRG neurons and spinal cord following SNL. These findings indicate that miR-30b is involved in the regulation of neuropathic pain by targeting Nav1.3, which might be a potential therapeutic target for neuropathic pain.

## Author Contributions

WZ and JC conceived the project, supervised all experiments, and wrote manuscript. SS and JS designed the project, researched data, and wrote manuscript. QZ, XR, and WC researched data and reviewed/edited manuscript. LL, QB, XC, BX, and JW reviewed/edited manuscript. All authors read and approved the final manuscript. SS and JS contributed equally to this study.

## Conflict of Interest Statement

The authors declare that the research was conducted in the absence of any commercial or financial relationships that could be construed as a potential conflict of interest.
